# Are zoonotic *Staphylococcus pseudintermedius* strains a growing threat for humans?

**DOI:** 10.1007/s12223-018-0615-2

**Published:** 2018-05-26

**Authors:** Wioletta Kmieciak, Eligia Maria Szewczyk

**Affiliations:** Department of Pharmaceutical Microbiology and Microbiological Diagnostics, Medical University of Łódź, Łódź, Poland

**Keywords:** *Staphylococcus pseudintermedius*, Colonization, Virulence, Companion animals, Skin microflora

## Abstract

*Staphylococcus pseudintermedius* is a species often isolated from animals, as a common element of their microbiota or an agent of infection, and from people associated with an animal habitat, including owners of home pets—dogs and cats. As with many other species, adaptation of these bacteria to the human body can occur, and they become important human pathogens. 59 *S. pseudintermedius* strains were investigated in this study to determine the factors contributing to human body colonization: inhibition growth of human skin residents isolated from human skin (*Staphylococcus epidermidis*, *Corynebacterium* spp., *Cutibacterium acnes* (formerly *Propionibacterium acnes*)), biofilm formation, and the presence of ten genes encoding infection-promoting features (including *ebpS*, *spsE*, *lukS*, *lukF*, *pvl*, *lip*, *hlgA*, *hlgB*). The ability of human skin to be colonized and the presence of genes that promote the development of skin infections showed the significant potential of the studied strains in their adaptation to the host. However, while a comparison of the characteristics of animal strains and those isolated from human infections does not allow us to claim that we are the witnesses of the speciation of a new human pathogen, it does indicate their gradual adaptation to the human organism.

## Introduction

*Staphylococcus pseudintermedius* belongs to the *Staphylococcus intermedius* group (SIG) which also comprises *Staphylococcus intermedius* and *Staphylococcus delphini* (Savini et al. 2013). These three coagulase-positive species are animal skin and mucous membrane commensals. *S. pseudintermedius* inhabits mainly companion animals such as pets (Jee et al. 2007; Kmieciak and Szewczyk 2017; Wladyka et al. 2015), but can also cause infections in animals (Międzobrodzki et al. 2010), most often otitis and skin infections (van Duijkeren et al. 2011). These bacteria have been also isolated from humans (Boost et al. 2011; Savini et al. 2014; Youn et al. 2014). They can be rare etiological factor of animal bite wound infections (Salina et al. 2015; Starlander et al. 2014; Windahl et al. 2015) but also more severe cases (Laurens et al. 2012; Riegel et al. 2011; Stegmann et al. 2010).

The transmission of microorganisms originally associated with animals to humans has been observed for centuries. The interspecies barriers separating animal species from humans are, e.g., tissue tropism, different cell receptors, differences between animal and human microbiota, health and immune status of them, different population dynamics and behavior of humans and animal, and abundance and spread of pathogens within and among species (Gortazar et al. 2014). After the adaptation process, animal pathogens gain more features that allow them to colonize human organisms and then cause serious diseases. Over time, they become recognized human pathogens (Wolfe et al. 2007). It is important to follow the changes of the original animal microorganisms in the context of current and future dangers to humans.

The aim of this study was to determine the features that could allow *S. pseudintermedius* to colonize human organism. The investigated strains were isolated from clinical specimens taken from pets and from humans. We examined the possibility of their settlement in the ecological niches alongside natural bacterial inhabitants of human skin, and evaluated the presence of genes that may play a role in the development of infections.

## Materials and methods

*S. pseudintermedius* strains causing infections, 40 from dogs and 2 from cats treated in veterinary clinics and 17 strains from the inpatients (7) and outpatients (10) of a hospital in Łódź, were investigated. These strains had been collected in the period of 1 year. All strains in both groups were isolated from skin lesions (4 human and 9 canine strains), infected wounds (4 human, 1 feline, and 6 canine strains), bedsores (1 human and 2 canine strains), external ear infections (7 human, 1 feline, and 19 canine strains), ophthalmia (1 human and 3 canine strains), and laryngitis (1 canine strain). Strains were identified by the genetic method developed by Sasaki et al. (Sasaki et al. 2010). The reference strain was *Staphylococcus pseudintermedius* PCM 2791.

### Phenotypic studies

Bacteria were grown on agar medium supplemented with 5% sheep blood. Hyaluronidase decomposition was studied using the method of Hart et al. (Hart et al. 2009). Biofilm formation was estimated in two colorimetric methods. The intensity of biofilm formation was assessed by crystalline violet (CV) staining according to Christensen et al. (Pompilio et al. 2015). The viability of the cells in the biofilm structure was tested by [2,3-bis(2-methoxy-4-nitro-5-sulfophenyl)-2H-tetrazolium-5-carboxanilide] (XTT) reduction method of Pettit et al. (Pettit et al. 2005). The ability to produce bacteriocin-like inhibitory substances (BLIS) was investigated using the direct method according to Balusek et al. (Balusek and Hájek 1985; Wysocki et al. 2011). The activity of these substances was evaluated against bacteria that were residents of healthy skin of young people. Ten strains from healthy skin belonging to the genera *Staphylococcus*, *Corynebacterium*, and *Cutibacterium* (formerly *Propionibacterium*) (Scholz and Kilian 2016) from our own collection were used for this study.

### Molecular research

In order to search for genes encoding virulence factors associated with colonization, primers recommended in literature and newly designed ones were used to PCR (polymerase chain reaction). The first ones were used to detect genes encoding: leukotoxin Luk-I S (*lukS*) and F (*lukF*) components, Panton-Valentine leucocidin (*pvl*), exfoliative toxin SIET (*siet*), toxic shock syndrome toxin (*tst*), enterotoxin A (*sea*), and enterotoxin C (*sec*) (Futagawa-Saito et al. 2004; Garbacz et al. 2013; Lina et al. 1999). The primers of the own project are shown in Table 1.

**Table 1:** Projected primers

Gene	Protein/toxin	Primer	Sequence (5′ → 3′)	Size of amplicons
*ebpS*	Elastin-binding protein	ebpS-F	AGACGCCACAGAAAAAGA	1040 bp
		ebpS-R	GCAGATTGACCTTGTTGA	
*spsE*	Fibrinogen-/fibronectin-binding protein	spsE-F	TTTCTCGTTTCTGGGCGT	1600 bp
		spsE-R	GCGTCTTCTGGTTATCGT	
*lip*	Lipase	lip-F	GGAAAAGCAGCAGAAAGAA	1601 bp
		lip-R	GGGTGCTGTGATGAAATA	
*hlgA*	γ-Hemolysin A component	hlgA-F	GTTCTTCCACTTATTACACC	718 bp
		hlgA-R	CACTTGTATCGCCTTTATC	
*hlgB*	γ-Hemolysin B component	hlgB-F	GGGGGGCTAAGTATAATGT	475 bp
		hlgB-R	GCGCCATTTGGTTTATGT	

The new primers were designed using the CLC Main Workbench 7.6 (QIAGEN), a program based on genome sequence analysis of strains deposited in Genbank:

- *Staphylococcus pseudintermedius* ED99 (NC_017568.1)—primers for the *ebpS*, *spsE*, and *lip* genes;
- *Staphylococcus pseudintermedius* HKU 10-03 (NC_014925.1)—primers for the *hlgA* and *hlgB* genes.

For PCR reactions with newly designed primers, conditions were as follows: initial denaturation at 94 °C for 2:30 min, 30 cycles (denaturation 0:30 min—94 °C; annealing 0:30 min—54 °C for detection of the genes *ebpS*, *spsE*, and *lip* or 56 °C for detection of genes *hlgA* and *hlgB*; elongation 1:00 min—72 °C) and final elongation 10:00 min—72 °C on a T Professional Basic Gradient Thermal Cycler (Biometra).

## Results

All 42 animal *S. pseudintermedius* strains were able to form biofilm structures that were defined as strong or moderate production (respectively 20 and 22). Most commonly, the formation of thick biofilm detected by the CV method was associated with a high metabolic activity resulting in significant XTT reduction ranging from 35 to 67%. Strains from humans did not differ from those of animals in this regard (Table 2).

**Table 2:** Features of *S. pseudintermedius* strains predisposing them to human body colonization

Biofilm forming		Human skin microbiota suppression			Pathogenicity gene presence (number of strains)									
CV method (number of strains)	Percentage of XTT reduction (median)	*Staphylococcus epidermidis* strains group:	*Corynebacterium* strains group:	*Cutibacterium acnes* strains group:	*ebpS*	*spsE*	*lukS* + *lukF*	*pvl*	*hlgA* + *hlgB*	*lip*	*siet*	*tst*	*sea*	*sec*
		- ZMF K22	- *Corynebacterium* sp. ZMF LP13	- ZMF P1										
		- ZMF K210	- *Corynebacterium* sp. ZMF LB136	*-* ZMF P3										
		- ZMF B84	- *C. tuberculostearicum* ZMF LA18	*-* ZMF R1										
				- ZMF R2										
Animal strains (42)														
42	42%	22 (1)*	7 (1)	3 (1)	41	40	4	1	42	40	42	0	0	2
		8 (2)	2 (3)	1 (3)										
		3 (3)	9 (21%)	5 (4)										
		33 (79%)		9 (21%)										
Human strains (17)														
17	50%	6 (1)	2 (1)	1 (4)	17	17	2	1	17	17	17	0	0	3
		7 (2)												
		1 (3)	2 (12%)	1 (6%)										
		14 (82%)												
All strains (59)														
59	46%	47	11	10	58	57	6	2	59	57	59	0	0	5

*The number of strains of the given group suppressed by *S. pseudintermedius* strains is given in brackets

Almost 80% of *S. pseudintermedius* strains exhibited an inhibitory effect on human skin microbiota, with the highest on epidermal staphylococci. Strains from animals were more effective than those from humans at inhibiting the growth of coryneform and *Cutibacterium* spp. This antibacterial activity was detected in 21% animal strains, while only 12% human strains were active against *Corynebacterium* spp. and 6% against *Cutibacterium* spp. The ability to inhibit several strains of one species or genus was more frequently observed among animal strains (Table 2, data in parentheses).

We also searched for genes encoding substances important for the colonization, spread, and toxicity of these staphylococci. The presence of extracellular matrix binding proteins of host tissues (*ebpS*, *spsE*) and the lipase enhancing the colonization of the skin, as well as the gene encoding the epidermolytic toxin, were common in *S. pseudintermedius* strains. In all strains, γ-hemolysin genes, *hlgA* and *hlgB*, were present. Few strains had both genes encoding leukotoxin Luk-I components (*lukS*, *lukF*). None of the tested strains had toxic shock syndrome toxin (*tst*) or enterotoxin A (*sea*) genes. Single strains had the gene of enterotoxin C (*sec*) (two from animals, three from humans) and Panton-Valentine leucocidin *pvl* genes (one from animal, one from humans) and showed hyaluronic acid degradation (eight from animals, two from humans) (Table 2). This is the first report on *pvl* gene detection in *S. pseudintermedius* strains.

## Discussion

Breaking the barrier protecting people from infections caused by animal strains must begin with the efficient colonization of human skin or mucous membranes. The tested *S. pseudintermedius* strains were capable of performing this process and they were able to effectively gain space on human skin by inhibiting the growth of bacteria that are its natural microbiota.

Our studies showed that *S. pseudintermedius* species has a potential for colonizing the human organism. Strains of this species possess phenotypical features and genes favoring staphylococcal pathogenicity to the skin. No significant differences were found between strains from animals or those that caused infections in humans that would indicate the adaptation of this species to a new host.

The acquisition of genes connected with human colonization may result from close contact between animals and their owners. This contact is certainly common in dogs and other domestic animals accompanying people as pets. Colonization of human skin also is interrelated with the ability of these staphylococci to form and live in biofilm structures. The acquisition of genes from human bacterial microbiota or pathogens can arise from rearrangement of genetic material and horizontal gene transfer (HTG) (Ambur et al. 2009). This may lead to the creation of new variants which are better adapted to a new host and their subsequent selection. We found a few strains that were better equipped than others.

Our results show the potential of the tested strains. *S. pseudintermedius* has a set of features that allow bacteria to infect humans and find a place among microbes on the skin of humans by inhibiting the growth of bacteria typical for this site.

Screening of the tested strains revealed the presence of toxin genes whose expression may contribute to the development of skin and subcutaneous tissue diseases. *S. aureus* Panton-Valentine leukocidin (PVL) is a factor of necrotic skin lesions (Barrio et al. 2006; Prévost et al. 1995). Its analogue in the SIG group is the Luk-I leukotoxin which exhibits a strong leukotoxic effect. The cytotoxic activity of bi-component leukotoxins and γ-hemolysin is manifested only when both components are synthesized (Szmigielski et al. 1998); both components was observed in most cases were the investigated strains possessed the genes.

SIET exfoliative toxin, which was detected in all of the tested strains, is involved in the etiopathogenesis of scaly and purulent skin infections (Terauchi et al. 2003). Lipase-producing strains can break down sebum, divesting the epidermis of an important antimicrobial agent, thus allowing effective colonization. Degradation of cell membrane lipids facilitates the spread of infection throughout the body (Hu et al. 2012). The gene *lip* encoding lipase was reported in almost all of the tested strains.

Our results indicate that a particular role in the pathogenesis of superficial infections may be played by the following: elastin binding protein EbpS, fibrinogen/fibronectin binding protein SpsE, lipase, exfoliative toxin SIET, and γ-hemolysin. The presence of these genes was commonly observed among *S. pseudintermedius* strains. Panton-Valentine leukocidin was detected only in two strains and it was the first time it was found in *S. pseudintermedius*. Our strains did not produce hyaluronidase, which enhances penetration through the deeper tissues of the host (Hart et al. 2013). Lack of toxic shock syndrome toxin (TSST-1) and staphylococcal enterotoxin genes which are superantigens reduce the chance of causing severe disease entities (Pinchuk et al. 2010). Enterotoxins A (SEA) and C (SEC) are a cause of food poisoning (STF). The toxin genes *tst* or *sea* were not present in the investigated *S. pseudintermedius* strains, although SEC is commonly found in other strains isolated from animals (Korpysa-Dzirba et al. 2012). The single occurrence of the *sec* gene in strains of this species from both the animals and humans indicates a potential risk of food poisoning.

It is difficult to determine how often *S. pseudintermedius* strains were previously isolated from clinical materials from humans and how frequently they are now isolated, as phenotypic identification of the species may be questionable. This is the largest study in Europe of *S. pseudintermedius* infections in humans. Most of the reports concern only single human strains although there is one study of 200 human isolates from south Japan (Bardiau et al. 2013). Many of the features we studied in human and animal strains were similar but some differences were found. It may be considered that this species is currently not a particular threat for humans but we showed the possibility of its settlement in the ecological niches alongside natural bacterial inhabitants of human skin and the presence of genes that may play a role in the development infections in humans. Further studies are needed to monitor change in phenotype and genotypic potential both of which may indicate transformation of *S. pseudintermedius* into a human pathogen.

## References

[CR1] Ambur OH, Davidsen T, Frye SA, Balasingham SV, Lagesen K, Rognes T, Tønjum T (2009). Genome dynamics in major bacterial pathogens. FEMS Microbiol Rev.

[CR2] Balusek J, Hájek V (1985). Antagonistic activities of coagulase-positive staphylococci. J Hyg Epidemiol Microbiol Immunol.

[CR3] Bardiau M, Yamazaki K, Ote I, Misawa N, Mainil JG (2013). Characterization of methicillin-resistant *Staphylococcus pseudintermedius* isolated from dogs and cats. Microbiol Immunol.

[CR4] Barrio MB, Rainard P, Prévost G (2006). LukM/LukF’-PV is the most active *Staphylococcus aureus* leukotoxin on bovine neutrophils. Microbes Infect.

[CR5] Boost MV, So SY, Perreten V (2011). Low rate of methicillin-resistant coagulase-positive staphylococcal colonization of veterinary personnel in Hong Kong. Zoonoses Public Health.

[CR6] Futagawa-Saito K, Sugiyama T, Karube S, Sakurai N, Ba-Thein W, Fukuyasu T (2004). Prevalence and characterization of leukotoxin-producing *Staphylococcus intermedius* in isolates from dogs and pigeons. J Clin Microbiol.

[CR7] Garbacz K, Zarnowska S, Piechowicz L, Haras K (2013). Pathogenicity potential of *Staphylococcus pseudintermedius* strains isolated from canine carriers and from dogs with infection signs. Virulence.

[CR8] Gortazar C, Reperant LA, Kuiken T, de la Fuente J, Boadella M, Martínez-Lopez B, Ruiz-Fons F, Estrada-Peña A, Drosten C, Medley G, Ostfeld R, Peterson T, VerCauteren KC, Menge C, Artois M, Schultsz C, Delahay R, Serra-Cobo J, Poulin R, Keck F, Aguirre AA, Henttonen H, Dobson AP, Kutz S, Lubroth J, Mysterud A (2014). Crossing the interspecies barrier: opening the door to zoonotic pathogens. PLoS Pathog.

[CR9] Hart ME, Hart MJ, Roop AJ (2009). Genotypic and phenotypic assessment of hyaluronidase among type strains of a select group of staphylococcal species. Int J Microbiol.

[CR10] Hart ME, Tsang LH, Deck J, Daily ST, Jones RC, Liu H, Hu H, Hart MJ, Smeltzer MS, Hart ME (2013). Hyaluronidase expression and biofilm involvement in *Staphylococcus aureus* UAMS-1 and its *sarA*, *agr* and *sarA agr* regulatory mutants. Microbiology.

[CR11] Hu C, Xiong N, Zhang Y, Rayner S, Chen S (2012). Biochemical and biophysical research communications functional characterization of lipase in the pathogenesis of *Staphylococcus aureus*. Biochem Biophys Res Commun.

[CR12] Jee H, Pakhrin B, Bae I-H, Shin N-S, Lee S-I, Yoo H-S, Kim D-Y (2007). Pyelonephritis associated with *Staphylococcus intermedius* in a Siberian tiger (*Panthera tigris altaica*). J Vet Med Sci.

[CR13] Kmieciak W, Szewczyk EM (2017). Coagulase-positive species of the genus *Staphylococcus* – taxonomy, pathogenicity. Postępy Mikrobiol.

[CR14] Korpysa-Dzirba W, Rola JG, Osek J (2012). Staphylococcal enterotoxins. Part I. Epidemiology and importance for public health. Życie Weter.

[CR15] Laurens C, Marouzé N, Jean-Pierre H (2012). *Staphylococcus pseudintermedius* and *Pasteurella dagmatis* associated in a case of community-acquired pneumonia. Médecine Mal Infect.

[CR16] Lina G, Pie Y, Godail-Gamot F, Peter M (1999). Involvement of Panton-Valentine leukocidin-producing *Staphylococcus aureus* in primary skin infections and pneumonia. Clin Infect Dis.

[CR17] Międzobrodzki J, Kasprowicz A, Białecka A, Jaworska O, Polakowska K, Władyka B, Dubin A (2010). The first case of a *Staphylococcus pseudintermedius* infection after joint prosthesis implantation in a dog. Pol J Microbiol.

[CR18] Pettit RK, Weber CA, Kean MJ, Pettit GR, Tan R, Franks KS, Horton ML (2005). Microplate Alamar Blue assay for *Staphylococcus epidermidis* biofilm susceptibility testing. Antimicrob Agents Chemother.

[CR19] Pinchuk IV, Beswick EJ, Reyes VE (2010). Staphylococcal enterotoxins. Toxins (Basel).

[CR20] Pompilio A, De Nicola S, Crocetta V, Guarnieri S, Savini V, Carretto E, Di Bonaventura G (2015). New insights in *Staphylococcus pseudintermedius* pathogenicity: antibiotic-resistant biofilm formation by a human wound-associated strain. BMC Microbiol.

[CR21] Prévost G, Cribier B, Couppié P, Petiau P, Supersac G, Finck-Barbançon V, Monteil H, Piemont Y (1995). Panton-Valentine leucocidin and gamma-hemolysin from *Staphylococcus aureus* ATCC 49775 are encoded by distinct genetic loci and have different biological activities. Infect Immun.

[CR22] Riegel P, Jesel-Morel L, Laventie B, Boisset S, Vandenesch F, Prévost G (2011). Coagulase-positive *Staphylococcus pseudintermedius* from animals causing human endocarditis. Int J Med Microbiol.

[CR23] Salina A, Machado GP, Guimarães F de F, Langoni H (2015). Microbial sensibility of *Staphylococcus* spp. isolated of goat milk with subclinical mastitis. Veterinária e Zootec.

[CR24] Sasaki T, Tsubakishita S, Tanaka Y, Sakusabe A, Ohtsuka M, Hirotaki S, Kawakami T, Fukata T, Hiramatsu K (2010). Multiplex-PCR method for species identification of coagulase-positive staphylococci. J Clin Microbiol.

[CR25] Savini V, Kosecka M, Marrollo R, Carretto E, Międzobrodzki J (2013). CAMP test detected *Staphylococcus delphini* ATCC 49172 beta-haemolysin production. Pol J Microbiol.

[CR26] Savini V, Paparella A, Serio A, Marrollo R, Carretto E, Fazii P (2014). *Staphylococcus pseudintermedius* for CAMP-test. Int J Clin Exp Pathol.

[CR27] Scholz CFP, Kilian M (2016). The natural history of cutaneous propionibacteria, and reclassification of selected species within the genus *Propionibacterium* to the proposed novel genera *Acidipropionibacterium* gen. nov., *Cutibacterium* gen. nov. and *Pseudopropionibacterium* gen. nov. J Syst Evolut Microbiol.

[CR28] Starlander G, Börjesson S, Grönlund-Andersson U, Tellgren-Roth C, Melhus Å (2014). Cluster of infections caused by methicillin-resistant *Staphylococcus pseudintermedius* in humans in a tertiary hospital. J Clin Microbiol.

[CR29] Stegmann R, Burnens A, Maranta CA, Perreten V (2010). Human infection associated with methicillin-resistant *Staphylococcus pseudintermedius* ST71. J Antimicrob Chemother.

[CR30] Szmigielski S, Sobiczewska E, Prévost G, Monteil H, Colin DA, Jeljaszewicz J (1998). Effect of purified staphylococcal leukocidal toxins on isolated blood polymorphonuclear leukocytes and peritoneal macrophages in vitro. Zentralblatt für Bakteriol.

[CR31] Terauchi R, Sato H, Hasegawa T (2003). Isolation of exfoliative toxin from *Staphylococcus intermedius* and its local toxicity in dogs. Vet Microbiol.

[CR32] van Duijkeren E, Kamphuis M, van der Mije IC, Laarhoven LM, Duim B, Wagenaar JA, Houwers DJ (2011). Transmission of methicillin-resistant *Staphylococcus pseudintermedius* between infected dogs and cats and contact pets, humans and the environment in households and veterinary clinics. Vet Microbiol.

[CR33] Windahl U, Bengtsson B, Nyman A-K, Holst BS (2015). The distribution of pathogens and their antimicrobial susceptibility patterns among canine surgical wound infections in Sweden in relation to different risk factors. Acta Vet Scand.

[CR34] Wladyka B, Piejko M, Bzowska M, Pieta P, Krzysik M, Mazurek Ł, Guevara-Lora I, Bukowski M, Sabat AJ, Friedrich AW, Bonar E, Międzobrodzki J, Dubin A, Mak P (2015). A peptide factor secreted by *Staphylococcus pseudintermedius* exhibits properties of both bacteriocins and virulence factors. Sci Rep.

[CR35] Wolfe ND, Dunavan CP, Diamond J (2007). Origins of major human infectious diseases. Nature.

[CR36] Wysocki P, Kwaszewska A, Szewczyk EM (2011). Influence of substances produced by lipophilic *Corynebacterium* CDC G1 ZMF 3P13 on the microorganisms inhabiting human skin. Med Dosw Mikrobiol.

[CR37] Youn J-H, Park YH, Hang’ombe B, Sugimoto C (2014). Prevalence and characterization of *Staphylococcus aureus* and *Staphylococcus pseudintermedius* isolated from companion animals and environment in the veterinary teaching hospital in Zambia, Africa. Comp Immunol Microbiol Infect Dis.

